# Genetic profiling of auto-inflammatory disorders in patients with periodic fever: a prospective study

**DOI:** 10.1186/1546-0096-12-S1-P83

**Published:** 2014-09-17

**Authors:** Carlo De Pieri, Josef Vuch, Anna Monica Bianco, Francesca Barbieri, Serena Pastore, Luca Ronfani, Sergio Crovella, Andrea Taddio, Giovanni Maria Severini, Aberto Tommasini

**Affiliations:** 1 institute For Maternal And Child Health Irccs Burlo Garofolo, Trieste, Italy; 2 department Of Medicine And Surgery And Health, University Of Trieste, Trieste, Italy; 3 institute For Maternal And Child Health Irccs Burlo Garofolo, Trieste, Italy ,Department Of Medicine And Surgery And Health, University Of Triestedepartment Of Medicine And Surgery And Health, University Of Trieste, Trieste, Trieste, Italy

## Introduction

Hereditary periodic fevers (HPF) are an emerging group of auto-inflammatory disorders. Although this group includes five well definite disorders, overlap of phenotypes can be often observed making the diagnosis more difficult. Although genetic diagnostics is currently available for different periodic fevers, many patients will require subsequent analyses for different genes. The selection of patients for genetic analysis is also not easy, despite scoring systems to assist the choice have been developed (Gaslini score).

## Objectives

We tested if a novel approach based on the simultaneous sequencing of HPF-related genes, can improve the diagnostics in this field.

## Methods

Patients consecutively referred to the unit of pediatric rheumatology of the IRCCS Burlo Garofolo from March 2012 to April 2013 for unexplained periodic fever since 1 year or more, still active at the time of recruitment. In particular we considered the following three groups: 1) patients already studied for a single candidate gene with negative results; 2) atypical pharyngitis, adenitis and aphthae (PFAPA) syndrome based on absent response to steroids or relapse after tonsillectomy; 3) other patients with periodic fever with multi-systemic involvement.

Structured chart was used to collect personal data and information about episode duration and clinical features, including symptoms, laboratory and imaging investigations results, response to treatments (steroids, colchicine).

In all subjects the simultaneous sequencing of MEFV, MVK, TNFRSF1A, NLRP3, NLRP12 was performed after fragment amplification in a single plate with specific primers with touch-down PCR.

## Results

A total of 43 patients were included in the study: 8 were previously evaluated for single genes (7 MVK, 2 NLRP3, 1 MEFV); 11 had atypical PFAPA (no response to glucocorticoids of tonsillectomy) ; 23 had periodic fever with multisystemic symptoms suspicious of no specific HPF.

According to the international consensus for the interpretations of genetic results, we could find: definitely causative mutations (V377I/V377I mutation in MVK; H304Y in NLRP12); low penetrance mutation (2 R92Q in TNFRSF1A); heterozygous high penetrance mutations (2 E148Q mutation in MEFV, 1 complex allele P369S-R408Q in MEFV, 1 V377I in MVK); single or multiple variants of unclear significance (overall 17, of which, 8 F402L variant in NLRP12, 5 Q703K variant in NLRP3). No variant in the five gene was found in 18 subjects.

Statistical analysis showed that the failure of glucocorticoids was significantly more frequent in subjects with any positive results to genetic analysis compared with subjects with negative genetic results.

## Conclusion

Simultaneous sequencing of multiple HPF-related genes can help diagnosing in few cases. In most cases a wide range of genetic abnormalities is observed, ranging from low penetrance mutations to complex genotypes with multiple variants in different genes.

## Disclosure of interest

None declared.

